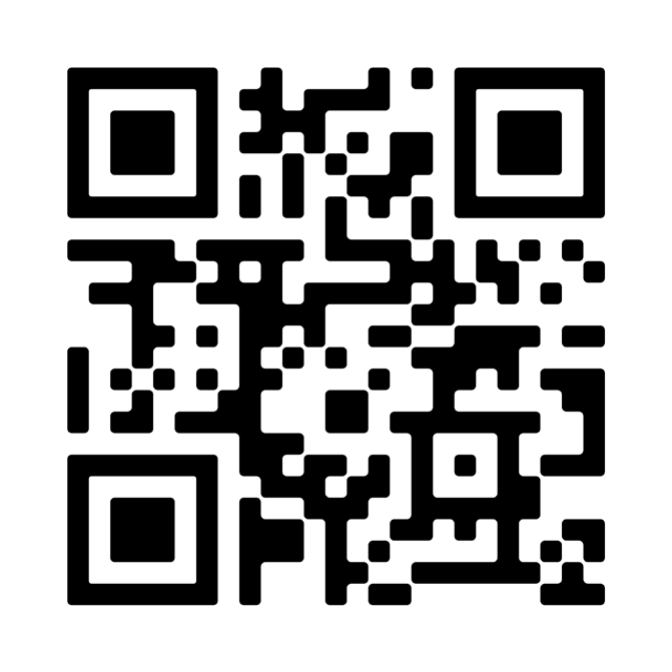# Complete bicorporeal uterus, double cervix, longitudinal obstructing vaginal septum: an integrated approach for one-stop diagnosis and ultrasound-guided endoscopic hymen-sparing treatment

**DOI:** 10.52054/FVVO.16.4.055

**Published:** 2024-12-27

**Authors:** F Bernardini, E Bonetti, F Pozzati, E La Fera, F Campolo, A Naldini, A.C. Testa, G Scambia, U Catena

**Affiliations:** Department of Woman and Child Health and Public Health, Fondazione Policlinico Universitario A Gemelli, IRCCS, Rome, Italy; Department of Clinical and Experimental Sciences, University of Brescia, ASST Spedali Civili, Italy; Università Cattolica del Sacro Cuore, Rome, Italy

**Keywords:** Uterine malformation, haematocolpos, bicorporeal uterus, obstructed hemivagina, Herlyn-Werner-Wunderlich syndrome, OHVIRA syndrome

## Abstract

**Background:**

Complete bicorporeal uterus, double cervix and obstructive longitudinal vaginal septum (classified as U3bC2V2 according to ESHRE/ESGE classification) is a rare congenital anomaly of the genital tract. This condition is typically associated with ipsilateral renal agenesis and is known as Herlyn-Werner-Wunderlich syndrome or OHVIRA (Obstructed HemiVagina and Ipsilateral Renal Anomaly) syndrome. The primary symptoms include dysmenorrhea and pelvic pain, which usually manifest after menarche due to haematocolpos in the obstructed hemivagina. Diagnosis is often challenging and frequently delayed. Early detection and surgical drainage of the haematocolpos are essential for symptom relief and prevention of complications. Various surgical approaches have been described, with vaginoplasty and septal resection being the recommended treatment.

**Objective:**

To propose a step-by-step demonstration with narrated video footage of an integrated approach for one-stop diagnosis and ultrasound-guided endoscopic hymen-sparing treatment in a patient with OHVIRA syndrome.

**Materials and Methods:**

We present the case of a 17-year-old virgo-intacta female who was referred to our institution due to dysmenorrhea, abnormal uterine bleeding and a right presumed ovarian endometrioma. A preoperative evaluation, including pelvic ultrasound and MRI, suspected a U3bC2V2 malformation, associated to a right haematocolpos and ipsilateral renal agenesis. The patient underwent a complete minimally invasive vaginoscopic resection of the obstructive longitudinal vaginal septum under transabdominal ultrasound guidance, using a 15Fr bipolar mini-resectoscope. The procedure successfully drained the haematocolpos and allowed visualisation of the right cervix, confirming the preoperative diagnosis.

**Results:**

The procedure was performed in our Digital Hysteroscopic Clinic (DHC) - CLASS Hysteroscopy - under general anaesthesia (with laryngeal mask), according to an ambulatory model of care. No complications occurred and the patient was discharged three hours after the procedure.

**Main outcomes:**

After 40 days, hysteroscopic office control revealed a normal vagina with double cervix and complete bicorporeal uterus (classified as U3bC2V0 according to ESHRE/ESGE classification), and the patient had complete relief of symptoms.

**Conclusion:**

The combined approach of one-stop diagnosis and ultrasound-guided minimally invasive vaginoscopic resection of the obstructive longitudinal vaginal septum, using a 15Fr bipolar mini-resectoscope, has proven to be an effective strategy. This approach leads to optimal surgical results without complications.

## Learning Objective

To propose a combined approach for a one-stop diagnosis and minimally invasive hymen-sparing treatment in a young symptomatic patient with obstructive longitudinal vaginal septum, double cervix and bicorporeal uterus, associated with ipsilateral renal agenesis.

## Introduction

Complete bicorporeal uterus, double cervix, and obstructive longitudinal vaginal septum, classified as U3bC2V2 according to ESHRE/ ESGE classification (Grimbizis et al., 2013), is a rare congenital anomaly of the genital tract. This condition is typically associated with ipsilateral renal agenesis and is known as Herlyn-Werner- Wunderlich syndrome or OHVIRA (Obstructed HemiVagina and Ipsilateral Renal Anomaly) syndrome. The first reported case of this anomaly dates back to 1922 (Purslow, 1922), it was later studied and extensively described by Herlyn, Werner and Wunderlich, who gave their name to this anomaly. Because of its rarity, the true incidence of the syndrome is not well known, but it is estimated that it may range from 0.1 to 3.8% in the female population (Kudela et al., 2021).

The aetiology is associated with the improper development of the Mullerian and the Wolffian ducts. Probably the absence of Wolff’s duct causes a lateral displacement of Muller’s duct that fails to fuse with the contralateral Muller’s duct, resulting in the bicorporeal uterus. In addition, the dislocated Muller’s duct fails to fuse properly with the urogenital sinus resulting in the obstructive longitudinal vaginal septum. On the other hand, abnormal development of the mesonephric duct results in renal anomalies (Kirplani et al., 2019).

The most common clinical features include dysmenorrhoea and lower abdominal pain, associated with haematocolpos. These symptoms typically manifest after menarche and are nonspecific, leading to delayed diagnosis. Diagnosis of this rare malformation can be challenging and often requires multiple diagnostic steps and techniques. In the past, the diagnosis was obtained by combining laparoscopy and hysteroscopy. However, magnetic resonance imaging (MRI) and ultrasound are now frequently used as alternatives to diagnostic laparoscopy. Advances in diagnostics have led to earlier detection and treatment, which can relieve symptoms and prevent complications such as endometriosis, adhesions, recurrent infections, infertility, and spontaneous miscarriages (Gungor et al, 2020).

Regarding treatment, only case reports and retrospective case series have been documented in the literature. The primary goal of treatment is to remove the obstruction, providing symptom relief and preventing long-term complications. Various surgical approaches have been described, including laparotomic, laparoscopic (Sleiman et al., 2019) and vaginal techniques. Among these, the vaginal approach is the most commonly used, with several surgical techniques reported in the literature (Borges et al., 2023), such as cold knife dissection (Altintaş, 1998), hysteroscopy with monopolar and bipolar resectoscope (Alumbreros et al., 2014; Piccini et al., 2015; Fascilla et al., 2020) and CO2 laser procedures (Tug et al., 2015).

Single stage vaginoplasty with drainage of the obstructed side and resection of the septum has emerged as the gold standard treatment (Gungor et al., 2020). Notably, Kriplani et al. (2019) and Fascilla et al. (2020) demonstrated that the vaginoscopic procedure with a hysteroscopic approach is both safe and effective in treating these patients. Additionally, Ludwin et al. (2018), in a case series involving four patients, showed that this management can also be applied to young, non- sexually active patients using a hymen-sparing approach.

In our video article, we propose a combined minimally invasive one-stop diagnosis and hysteroscopic ultrasound-guided hymen-sparing treatment for a patient with OHVIRA syndrome, using a 15Fr bipolar mini-resectoscope.

## Case description

A 17-year-old, not yet sexually active girl, was referred to our clinic - Fondazione Policlinico Gemelli IRCCS of Rome, Italy – due to dysmenorrhea, abnormal uterine bleeding and a suspected right ovarian endometrioma. Transrectal and transabdominal 2D and 3D ultrasound examinations, performed at our ultrasound centre - Class Ultrasound OMIC-, revealed the presence of a bicorporeal uterus with two cervices, a 6 cm collection on the right side of the vagina and a right- sided renal agenesis. The pelvic MRI, requested by our ultrasonographers, confirmed the presence of a double uterus and cervices, associated with a right haematocolpos and ipsilateral renal agenesis with mild compensatory hypertrophy of the left kidney, raising suspicion of OHVIRA syndrome.

We proposed a stepwise demonstration with narrated video footage of an integrated approach for a one-stop diagnosis and ultrasound-guided hysteroscopic hymen-sparing treatment, using a 15Fr bipolar mini-resectoscope.

The surgery was accepted by the patient and her parents and was performed in our Digital Hysteroscopic Clinic (DHC) - CLASS Hysteroscopy -, under general anaesthesia, using laryngeal mask, according to an ambulatory model of care (Carugno et al., 2021; Campo et al., 2018).

A complete minimally invasive hysteroscopic approach, guided by transabdominal ultrasound, was performed by an expert operator (UC), as follows:

1. Vaginoscopic access using a 5 mm Bettocchi hysteroscope (Karl Storz, Tuttlingen, Germany) revealed a bulge on the upper two-thirds of the right vaginal wall due to haematocolpos. The normal left cervix provided access to the left hemiuterus, with visualisation of the ipsilateral tubal ostium.
2. 5 Fr cold scissors were introduced in the operative channel of the Bettocchi hysteroscope. A careful vaginoscopic incision of the right vaginal wall was obtained under transabdominal ultrasound guidance, in order to access the right hemivagina.
3. Drainage of the right haematocolpos allowed visualisation of a normal right cervix, confirming the preoperative diagnosis.
4. Access to the right uterine hemiuterus, with visualisation of the ipsilateral tubal ostium. No communication was found at the level of the cervices or uterine hemicavities.
5. Complete resection of the vaginal septum was performed using a Collins loop of the 15 Fr bipolar miniresectoscope (Karl Storz, Tuttlingen, Germany), reaching the plane of the two cervices.

This one-stop ultrasound and hysteroscopic integrated approach allowed confirmation of the preoperative diagnosis of U3bC2V2 and the ability to perform vaginoplasty with a hymen- sparing approach. The patient received no intra- or postoperative antibiotic prophylaxis.

Double cervix and complete bicorporeal uterus were left unmodified because no surgical indications were recommended (Ludwin et al., 2013).

## Results

No complications occurred during and after the procedure. The patient was discharged three hours after the procedure.

The hysteroscopic office control, performed 40 days after procedure, revealed a normal vagina without residual vaginal septum, two normal cervices and a complete bicorporeal uterus (classified as U3bC2V0 according to ESHRE/ ESGE classification). The patient for the first time had a complete relief of symptoms.

## Discussion

In this video article we present a step-by-step demonstration using video footage of an integrated approach for the diagnosis and ultrasound-guided endoscopic hymen-sparing resection of longitudinal obstructive vaginal septum in a young symptomatic patient with U3bC2V2 anomaly (Grimbizis et al., 2013), associated to ipsilateral renal agenesis (OHVIRA syndrome). To our knowledge, this is the first time that a 15 Fr bipolar mini-resectoscope was used to treat this condition.

The strength of our technique lies in the ability to diagnose and treat a complex malformation on the same calendar day, avoiding multiple steps. This type of management, widely used in the treatment of other congenital anomalies of the female genital tract, minimises unnecessary delays, simplifies care and reduces patient stress (Pozzati et al., 2023).

Another point of strength is that our approach can be proposed not only to virgo-intacta women but to all young women in the post-pubertal phase, where the particularly narrow vagina may limit the visibility and accuracy of the specular examination.

The surgery was concluded in a one-stop endoscopic procedure under transabdominal ultrasound guidance, according to an ambulatory model of care (Carugno et al., 2021). The minimally invasive approach and ambulatory model of care are additional strengths of our treatment, enabling the patient to be discharged 3 hours after surgery.

Transabdominal ultrasound guidance identified the optimal site for vaginal septum incision, allowing for blood drainage and avoiding false paths. As demonstrated in the literature, ultrasound guidance has proven to be a valuable tool to guide vaginal septal incision, especially in small septa, where the risk of following false paths is higher. Recently, not only transabdominal but also transrectal approaches with or without aqueous contrast have been proposed. (Ludwin et al., 2018).

Although only case series and case reports are available in the literature, it is known that the primary goal of treating this malformation is removal of the vaginal septum. Traditionally, the procedure was performed vaginally through incision, drainage and marsupialisation of the vaginal septum; however, it is preferable not to be performed in patients who are not yet sexually active, as it would result in tearing of the hymen. Instead, hysteroscopy performed by an expert operator allows for a minimally invasive treatment keeping the hymen intact.

Early resection of the vaginal septum is an effective method to manage cases with haematocolpos. However, hemihysterectomy was proposed for patients with severe complications, such as endometriosis, adnexal damage and extensive pelvic adhesions (Bolonduro et al., 2015).

Early diagnosis of this complex malformation is important to avoid long-term complications. A major limitation of our paper is that it is a single- case representation, and more cases should be gathered to describe appropriate results.

## Conclusions

A combined one-stop diagnosis and ultrasound- guided minimally invasive endoscopic resection of the obstructive longitudinal vaginal septum, using a 15Fr bipolar miniresectoscope, can be safely performed in a digital hysteroscopic clinic with optimal surgical results and without complications, even in patients who are virgo intact. Early and accurate diagnosis of congenital Müllerian anomalies is crucial in identifying patients who may benefit from surgical intervention. This allows for symptom relief and complication prevention. However, further studies are needed to standardise the technique and evaluate the potential long-term clinical and fertility outcomes of these patients.

## Video scan (read QR)

https://vimeo.com/1007985686/554dc863c1?share=copy

**Figure:**